# Prognostic Nutritional Index as a Predictor of Treatment-Related Toxicity in Head and Neck Cancer Patients Undergoing Chemoradiotherapy

**DOI:** 10.7759/cureus.113096

**Published:** 2026-07-21

**Authors:** Vyshnavi Katta, S.M. Azeem Mohiyuddin, Monesha Baskaran

**Affiliations:** 1 Otolaryngology and Head and Neck Surgery, Sri Devaraj Urs Academy of Higher Education and Research, Kolar, IND

**Keywords:** chemotherapy-related toxicity, head and neck cancer, mucositis, prognostic nutritional index, treatment toxicity, weight loss

## Abstract

Background

The Prognostic Nutritional Index (PNI) is a straightforward marker that reflects nutritional and inflammatory status, derived from serum albumin and lymphocyte count. The significance of predicting treatment-related toxicity in patients with head and neck cancer (HNC) receiving radiotherapy in conjunction with chemotherapy continues to hold clinical importance. This study aimed to assess the predictive capability of baseline PNI regarding severe acute and late radiation-induced toxicities (mucositis, dysphagia, xerostomia, weight loss)in HNC patients undergoing radiotherapy with either induction or concurrent chemotherapy.

Methodology

This retrospective cohort study comprised 150 patients with HNC who received treatment at a tertiary center from January 2021 to December 2025. The PNI was determined utilizing Onodera’s formula, and patients were classified into low (<49.0) and high (≥49.0) PNI categories. The outcomes evaluated comprised severe acute toxicity (Common Terminology Criteria for Adverse Events, version 4.0 grade ≥3), weight loss exceeding 10% during treatment, and late toxicities (grade ≥2). Analyses using multivariable logistic and Cox regression were conducted.

Results

The median PNI was recorded at 49.0, with 50% of participants classified as having low PNI. Severe acute toxicity was observed in 64% of patients, whereas 20% experienced notable weight loss. Patients with low PNI experienced greater weight loss (32% compared to 8%) and a heightened risk after adjustment (odds ratio = 4.12). A low PNI was similarly linked to the occurrence of late mucositis (hazard ratio = 1.82).

Conclusions

A low baseline PNI serves as a predictor for treatment-related toxicity and may assist in identifying high-risk HNC patients who would benefit from early nutritional intervention.

## Introduction

Head and neck cancers (HNCs), especially oral squamous cell carcinoma, represent a significant health challenge in India, comprising nearly 30% of all cancer cases and ranking sixth worldwide, with a greater incidence in low- and middle-income nations [1]. Malnutrition impacts 30-50% of patients with HNC at the time of diagnosis and deteriorates during treatment as a result of tumor burden and the toxicities associated with therapy [2]. Radiotherapy and chemotherapy frequently lead to mucositis, dysphagia, and weight loss, which negatively affect treatment tolerance and outcomes [3]. The Prognostic Nutritional Index (PNI), which is calculated using serum albumin (g/dL) and lymphocyte count (cells/mm³), serves as an indicator of nutritional and immune status and has the potential to forecast treatment-related toxicity [4,5]. Low PNI indicates a state of malnutrition and systemic inflammation, both of which may impair treatment tolerance and tissue recovery. PNI has shown prognostic significance across several malignancies, including lung, colorectal, breast, and cervical cancers [2]. In HNC, low PNI has been associated with poor survival in both surgically and non-surgically treated patients.

## Materials and methods

Study design and setting

This was a retrospective, single-center, observational cohort study (analytical, non-interventional design) conducted at a tertiary care hospital, analyzing the medical records of patients who received treatment from January 2021 to December 2025. The study period (treatment delivery window) spanned 60 months; data abstraction and analysis were undertaken after a minimum of six months’ follow-up had elapsed for the last enrolled patient to allow ascertainment of late toxicity. The Institutional Ethics Committee approved the study (reference number: SDUAHER/R&D/CEC/SDUMC-PG/320/NF/-2025-26), and the study was conducted in accordance with the Declaration of Helsinki. As a retrospective record-based study, individual informed consent was waived by the Ethics Committee, and patient confidentiality was maintained throughout data abstraction and analysis.

Sampling technique

A non-probability, consecutive sampling was employed by including all eligible patients treated during the study period until the predefined study period ended. All eligible patients with head and neck squamous cell carcinoma who underwent definitive chemoradiotherapy at the study center during the study period and who satisfied the inclusion and exclusion criteria were enrolled sequentially, without random selection, until the study period closed. This sampling approach is appropriate for a retrospective cohort design drawing on a finite, identifiable institutional population, though it carries an inherent risk of selection bias, which is addressed in the Limitations section.

Study population

The study included consecutive adult patients (≥30 years) diagnosed with histologically confirmed squamous cell carcinoma of the oropharynx, hypopharynx, larynx, or nasopharynx who underwent definitive external beam radiotherapy (EBRT) alongside induction or concurrent chemotherapy.

Inclusion and exclusion criteria

The study included individuals aged 30 years and older with histologically confirmed squamous cell carcinoma of the head and neck who underwent definitive radiotherapy alongside either induction or concurrent chemotherapy, and who had a thorough and documented clinical evaluation of treatment-related toxicity during and following therapy. Further, patients with a documented baseline serum albumin and total lymphocyte count, baseline anthropometric measurements (weight and height), complete treatment records, and a minimum follow-up period of six months were considered for inclusion.

We excluded those who had undergone previous radiotherapy or surgical procedures in the head and neck area, those with recurrent disease or a second primary malignancy, those with distant metastasis at the time of treatment, those in whom radiotherapy was administered as a standalone treatment, without the inclusion of concurrent or induction chemotherapy, and those with insufficient clinical or laboratory data necessary for the calculation of the PNI. We also excluded those with active uncontrolled infection, autoimmune disease, or chronic inflammatory illness, and concurrent corticosteroid or immunosuppressive therapy at baseline, as these conditions independently alter serum albumin and lymphocyte count and would confound PNI estimation.

Sample size

This was a retrospective study using a convenience sample comprising all consecutive eligible patients treated at the center during the defined study period; no a priori sample size calculation was performed, as is customary for retrospective cohort designs that analyze the full available institutional dataset rather than a recruited sample. With 150 patients (75 per PNI group), the study had adequate power to detect the effect sizes that were observed (e.g., adjusted odds ratio (OR) of 4.12 for weight loss, adjusted hazard ratio (HR) of 1.82 for late mucositis); post hoc power for the weight-loss outcome, based on the observed event rates (32% vs 8%) and a two-sided alpha of 0.05, exceeded 90%, while power to detect the smaller effect on late mucositis (16.0% vs. 6.7%) was more modest (approximately 60-65%), which should be considered when interpreting the borderline confidence interval (CI) for that outcome (95% CI = 1.04-3.18). The non-significant association with overall severe acute toxicity may, in part, reflect inadequate power rather than a true absence of effect, which is noted as a limitation.

Confounders

The following variables were pre-specified as potential confounders, based on biological plausibility and prior literature, and were entered into the multivariable logistic and Cox regression models a priori: age, performance status (Eastern Cooperative Oncology Group), tumor site, disease stage (American Joint Committee on Cancer (AJCC) 8th edition), smoking status, and baseline body mass index (body mass index (BMI)). Radiotherapy technique/dose and chemotherapy regimen (induction vs. concurrent) were also recorded and examined as covariates, given their established influence on acute toxicity. Variables were retained in the final models regardless of statistical significance at univariate screening, consistent with a confounder-driven (rather than purely statistical) model-building strategy.

Data collection

Clinical and demographic data were obtained from institutional medical records. The collected variables included age, sex, tumor site, disease stage, treatment specifics (radiotherapy dosage, chemotherapy regimen), baseline nutritional parameters (serum albumin, lymphocyte count), and follow-up data. BMI was calculated using baseline weight and height. Diagnostic confirmation of malignancy in all patients followed the World Health Organization (WHO) classification of head and neck tumors [6], and disease extent was staged according to the AJCC staging manual, 8th edition [7].

Assessment of the Prognostic Nutritional Index

The PNI was calculated using Onodera’s formula [4]: PNI = (10 × serum albumin, g/dL) + (0.005 × total lymphocyte count/mm³). This formula, originally derived for gastrointestinal surgical patients [4], has subsequently been validated in HNC cohorts undergoing chemoradiotherapy [5]. Patients were classified into low and high PNI groups using the cohort’s own median value (49.0) as the cutoff (low PNI <49.0; high PNI ≥49.0), in keeping with the data-driven cutoff approach reported in comparable HNC studies [5] in the absence of a single universally validated threshold for this population.

Details of treatment

All patients underwent definitive EBRT in accordance with institutional protocols, either as a standalone treatment with induction chemotherapy or in conjunction with chemotherapy. Radiotherapy dosing schedules and chemotherapy regimens were implemented in accordance with established clinical guidelines. Radiotherapy was delivered to a total dose range consistent with definitive intent (66-70 Gy in conventional fractionation, or biologically equivalent altered fractionation schedules), and concurrent chemotherapy followed a platinum-based regimen, with regimen type and cycle number recorded as covariates.

Outcome assessment

The main outcome was the incidence of treatment-related toxicity, encompassing both acute and late radiation-induced toxicities. The toxicities of interest comprised mucositis, dysphagia, xerostomia, and notable weight loss. Toxicity grading was evaluated utilizing established criteria, specifically the Common Terminology Criteria for Adverse Events, version 4.0 (CTCAE v4.0) [8]. Acute toxicity was defined as that occurring during or within 90 days of completing radiotherapy, and late toxicity as that occurring or persisting beyond 90 days, in keeping with the standard Radiation Therapy Oncology Group (RTOG) convention [9]. Patients were monitored for a minimum period of six months following treatment completion. Clinical records were examined to document treatment-related toxicities and disease status throughout the follow-up period.

Statistical analysis

Data analysis was performed using SPSS version 26.0 (IBM Corp., Armonk, NY, USA). Continuous variables were first assessed for normality using the Shapiro-Wilk test and visual inspection of histograms/Q-Q plots. Normally distributed continuous variables (e.g., age, BMI) are reported as mean ± standard deviation and were compared between PNI groups using the independent-samples t-test; non-normally distributed continuous variables (e.g., PNI, follow-up duration) are reported as median with interquartile range (IQR) and were compared using the Mann-Whitney U test, as these data did not satisfy the normality assumption required for parametric testing. Categorical variables are presented as frequencies and percentages and were compared between low- and high-PNI groups using the chi-square test (or Fisher’s exact test where any expected cell count was <5), as appropriate for comparing proportions between two independent groups.

The choice of regression model was determined by outcome type and the presence of time-to-event data. For binary outcomes assessed at a fixed endpoint (severe acute toxicity ≥3 and weight loss >10%, both ascertained as present/absent during the treatment course), multivariable binary logistic regression was used to estimate adjusted OR with 95% CIs, as logistic regression is the standard approach for modelling a dichotomous outcome against multiple categorical and continuous predictors without requiring distributional assumptions on the predictors themselves. For late mucositis, which was a time-to-event outcome subject to variable follow-up duration and censoring (patients followed for differing lengths of time up to a median of 36 months), multivariable Cox proportional hazards regression was used to estimate adjusted HRs, as this method correctly accounts for censored observations and differential follow-up time, which a logistic model would ignore. The proportional hazards assumption was assessed using Schoenfeld residuals. Overall survival was estimated using the Kaplan-Meier method, with between-group comparison by the log-rank test, consistent with standard time-to-event analysis for a right-censored outcome.

All multivariable models (logistic and Cox) were adjusted a priori for the pre-specified confounders listed above (age, performance status, tumor site, stage, smoking status, and BMI), selected based on biological plausibility and prior literature rather than stepwise statistical selection to minimize the risk of overfitting and post hoc model selection bias inherent to a retrospective dataset of this size.

A p-value of less than 0.05 was deemed statistically significant for all tests, which were two-tailed. Given the modest sample size and the number of outcomes examined, the late mucositis association (95% CI = 1.04-3.18) should be interpreted with appropriate caution, as reflected in the Limitations section.

## Results

Characteristics of patients

A total of 150 patients satisfied the inclusion criteria and were incorporated into the analysis. The median age was 58 years (range = 50 ± 30years), with a majority of males (108; 72%). The main locations of the tumors were the oropharynx (48%), hypopharynx/larynx (30%), and nasopharynx (22%). The majority of patients exhibited advanced disease, with 64% categorized as stage III-IV (stage III: 36%; stage IV: 28%).

The mean baseline BMI was 25.1 ± 3 kg/m², with an IQR of 23.0 to 28.4. The median PNI was recorded at 49.0, with an IQR of 45.0 to 52.5. The patients were evenly allocated into two groups: low PNI (<49.0) and high PNI (≥49.0), with each group consisting of 75 participants. Baseline characteristics were comparable between the groups, as detailed in Table 1.

**Table 1:** Baseline characteristics by PNI group. BMI = body mass index; PNI = Prognostic Nutritional Index; IQR = interquartile range

Characteristic	Overall (N = 150)	Low PNI (N = 75)	High PNI (N = 75)
Age ≥ 60 years	68 (45%)	36 (48%)	32 (43%)
Male sex	108 (72%)	56 (75%)	52 (69%)
Stage III–IV	96 (64%)	50 (67%)	46 (61%)
Median BMI (kg/m²)	25.1	24.6	25.6
Median PNI (IQR)	49.0 (45.0–52.5)	45.8 (43.0–47.9)	52.4 (50.1–54.3)

Acute toxicity and weight reduction

A total of 96 (64.0%) patients encountered at least one instance of severe acute toxicity (grade ≥3). The most commonly observed events included mucositis at 38.7%, dermatitis at 13.3%, and weight loss exceeding 10% at 20.0%.

Notable weight loss (>10%) was observed more frequently in the low PNI group in comparison to the high PNI group (32.0% vs. 8.0%). In univariate analysis, a low PNI was linked to an increased risk of significant weight loss (OR = 5.41; 95% CI = 2.06-14.21). The association continued to show statistical significance following multivariable adjustments for various potential confounders, such as age, performance status, tumour site, stage, smoking status, and BMI (adjusted OR = 4.12; 95% CI = 1.52-11.18; p=0.005). Nonetheless, no notable correlation was detected between baseline PNI and overall severe acute toxicity (adjusted OR = 1.35; 95% CI = 0.78-2.34; p = 0.29).

Delayed toxicity and prognosis

At a median follow-up of 36 months (IQR = 22-60), 60 (40%) patients experienced at least one late toxicity of grade ≥2. The predominant late toxicities observed included xerostomia at 22.7%, hypoacusia at 13.3%, and late mucositis at 11.3%. Late mucositis was observed more frequently in patients exhibiting a low PNI (16.0%) in comparison to those with a high PNI (6.7%). In the multivariable Cox regression analysis, a low PNI was found to be independently linked to a heightened risk of late mucositis (adjusted HR = 1.82; 95% CI = 1.04-3.18; p = 0.036).

The three-year survival rate was 70%. Patients who experienced a weight loss greater than 10% demonstrated significantly lower survival rates compared to those without substantial weight loss (58% vs. 76%; log-rank p = 0.02).

Multivariable analysis

The key associations identified through multivariable analysis are presented in Table 2. A low baseline PNI was found to be independently linked to a heightened risk of substantial weight loss and late mucositis; however, it did not show a significant correlation with overall severe acute toxicity.

**Table 2:** Key associations (multivariable). PNI = Prognostic Nutritional Index; OR = odds ratio; HR = hazard ratio; CI = confidence interval

Outcome	Adjusted estimate	95% CI (p-value)
Weight loss >10% (low vs. high PNI)	OR = 4.12	1.52–11.18 (p = 0.005)
Late mucositis (low vs. high PNI)	HR = 1.82	1.04–3.18 (p = 0.036)
Overall severe acute toxicity (low vs. high PNI)	OR = 1.35	0.78–2.34 (p = 0.29)

**Figure 1:**
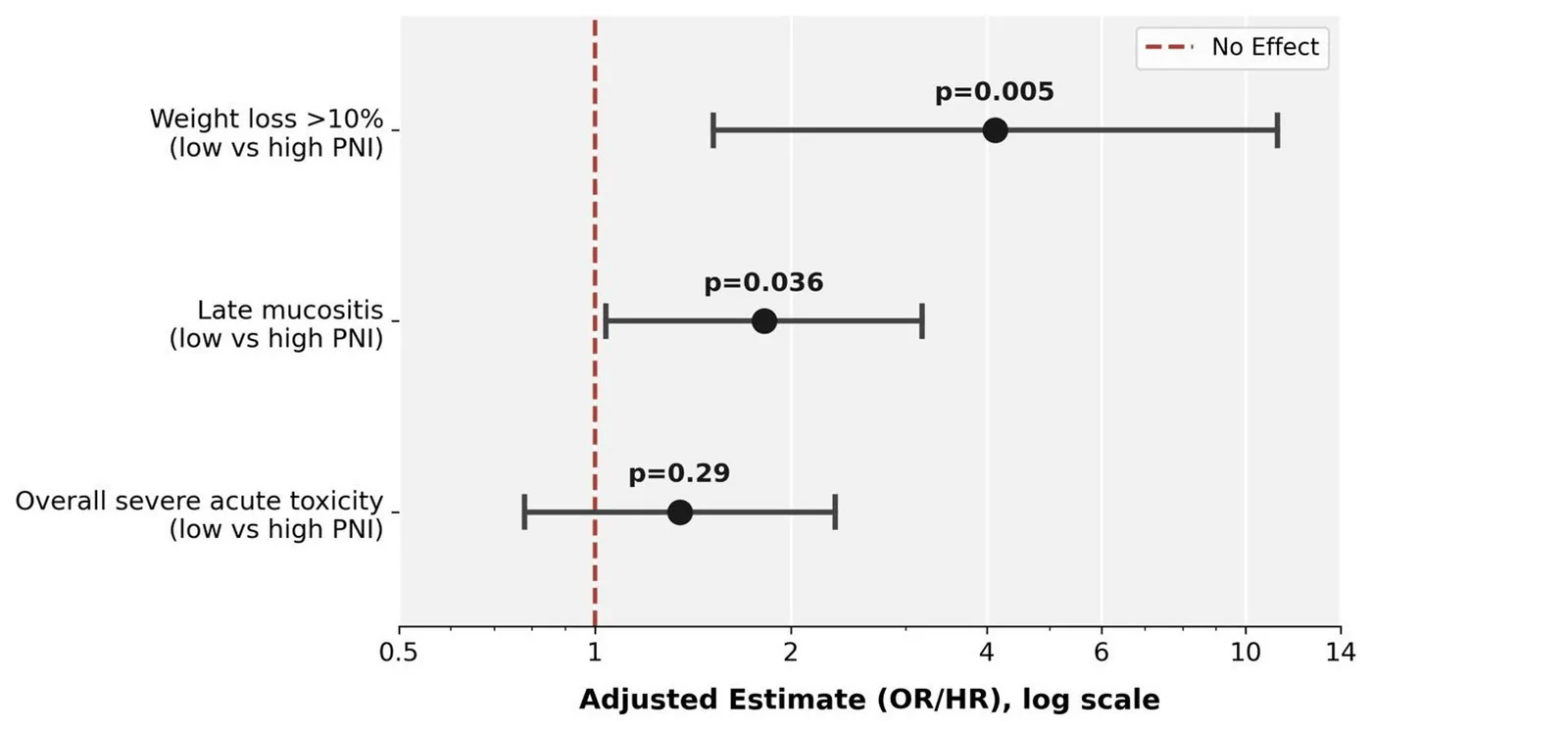
Forest plot of adjusted estimate with 95% CIs. PNI = Prognostic Nutritional Index; CI = confidence interval

The forest plot (Figure 1) presents adjusted effect estimates accompanied by 95% CIs. The association between low PNI and weight loss exceeding 10%, as well as late mucositis, was significant, as evidenced by estimates greater than 1 and CIs that did not include unity. The relationship between PNI and overall severe acute toxicity did not reach statistical significance, as the CI crossed the line of no effect.

## Discussion

In this study, we assessed the role of the PNI as a predictor of treatment-related toxicity in patients with HNC receiving definitive radiotherapy combined with chemotherapy. The results indicate that a low baseline PNI is significantly linked to a heightened risk of clinically relevant weight loss and late mucositis, whereas no significant correlation was found with overall severe acute toxicity.

Malnutrition is commonly observed in HNC patients and is recognized to deteriorate during treatment as a result of tumor burden and therapy-related toxicities, including mucositis and dysphagia [10]. In our cohort, 20% of patients experienced weight loss exceeding 10%, with a significantly higher incidence observed in the low PNI group. This aligns with earlier research suggesting that inadequate baseline nutritional status makes patients more susceptible to treatment-related catabolism and reduced intake [11]. A similar magnitude of effect was reported by Arora et al. in an Indian cohort of HNC patients undergoing chemoradiotherapy, in which a low pre-treatment PNI was independently associated with greater than 10% weight loss during treatment, supporting the generalizability of this association across populations [12]. The significant correlation identified between low PNI and weight loss in our study underscores its potential as a surrogate marker for nutritional reserve and systemic inflammation.

Our findings indicate that a low PNI is independently linked to a heightened risk of late mucositis. This can be elucidated by the interaction among malnutrition, immune suppression, and compromised tissue repair mechanisms. Serum albumin indicates protein reserves and systemic inflammation, while lymphocyte count signifies immune competence; collectively, they affect the body’s capacity to recover from radiation-induced mucosal injury [13]. Comparable results have been observed by Fanetti et al., who indicated that a low pre-treatment PNI was a predictor of both weight loss and late mucosal toxicity in patients undergoing chemoradiotherapy [5]. A subsequent meta-analysis by Wang et al., pooling data from HNC chemoradiotherapy cohorts, likewise found low PNI to be associated with a higher composite incidence of severe acute and late mucosal toxicity, reinforcing the biological plausibility of this relationship across diverse populations and treatment protocols [14].

Notably, baseline PNI did not show a significant correlation with overall severe acute toxicity in our study. This indicates that acute toxicities are likely more directly affected by treatment-related factors, including radiation dose, technique, and concurrent chemotherapy, rather than solely by baseline nutritional status. The influence of PNI on late toxicities underscores its significance in long-term outcomes and survivorship. This pattern is consistent with the findings of Lee et al., who reported that nutritional indices were stronger predictors of late, rather than acute, radiation-induced toxicity in nasopharyngeal carcinoma, attributing this to the dependence of acute mucosal injury on direct radiation dosimetry rather than systemic nutritional reserve [15].

The prognostic significance of PNI has been extensively documented in numerous malignancies, such as lung, colorectal, and breast cancers, indicating that a low PNI correlates with diminished survival rates and a higher incidence of complications. Okamoto et al. similarly demonstrated that low PNI was an independent predictor of overall survival in esophageal squamous cell carcinoma patients undergoing chemoradiotherapy, a tumor biologically and therapeutically analogous to HNC [16]. In HNC specifically, prior research has shown its correlation with survival outcomes; our findings build upon this evidence by highlighting its significance in forecasting treatment tolerance and toxicity.

From a clinical standpoint, PNI serves as a straightforward, cost-effective, and readily accessible biomarker obtained from standard laboratory parameters [17]. Timely recognition of patients exhibiting low PNI can facilitate focused interventions, including nutritional optimisation, dietary counselling, and enhanced monitoring throughout the treatment process. These strategies may assist in alleviating toxicity, minimizing treatment interruptions, and potentially enhancing overall outcomes. This is supported by the systematic review of Langius et al., which found that structured nutritional intervention during (chemo)radiotherapy improved nutritional status and, in several included studies, treatment completion rates in HNC patients [18].

Limitations

The retrospective design may lead to selection bias, and the use of consecutive, non-probability sampling from a single tertiary center may limit generalizability to other practice settings. While the sample size is sufficient to detect the larger effect observed for weight loss, it provides only modest power for the late mucositis outcome, as discussed in the Methods, restricting the generalizability of the findings. Furthermore, differences in treatment protocols and supportive care strategies may have impacted toxicity outcomes. The use of a cohort-derived (rather than externally validated) PNI cutoff also limits direct comparability with other studies until a population-specific threshold is prospectively validated. It is essential to conduct prospective, multicenter studies to confirm these findings and to establish standardized PNI cutoff values for clinical application.

## Conclusions

This study found that a low baseline PNI was independently linked to an increased risk of significant weight loss and late mucositis in patients with HNC receiving definitive chemoradiotherapy. Nonetheless, PNI did not show a significant association with overall severe acute toxicity. The findings underscore the significance of baseline nutritional and immunological status in shaping treatment tolerance and late toxicity outcomes. PNI, being a straightforward and easily accessible biomarker, could be a valuable addition for risk stratification in standard clinical practice.
